# Intrinsic persistent firing in CA1 encodes elapsed time across behaviorally relevant scales

**DOI:** 10.64898/2025.12.10.693586

**Published:** 2025-12-13

**Authors:** Sara Zomorodi, Beate Knauer, Yacine Brahimi, Antonio Reboreda, Motoharu Yoshida, Zoran Tiganj

**Affiliations:** 1 Department of Computer Science, Indiana University, Bloomington, IN; 2 International Graduate School of Neuroscience (IGSN), Ruhr University Bochum, Bochum, Germany; 3 Functional Architecture of Memory, Leibniz Institute for Neurobiology (LIN), Magdeburg, Germany; 4 German Center for Neurodegenerative Diseases (DZNE), Magdeburg, Germany; 5 Medical Faculty, Otto-von-Guericke Universität (OvGU), Magdeburg, Germany; 6 Center for Behavioral Brain Sciences (CBBS), Magdeburg, Germany; 7 Department of Psychological and Brain Sciences, Indiana University, Bloomington, IN

**Keywords:** Persistent firing, working memory, time perception, temporal tuning, carbachol, patch-clamp techniques

## Abstract

The ability to encode and maintain temporal relationships is crucial for learning, predicting, and forming episodic memories. While hippocampal time cells and entorhinal temporal context cells are well-established *in vivo*, it remains unclear whether single neurons can sustain representations of elapsed time over multi-second intervals, independent of synaptic drive. Here, using whole-cell patch-clamp recordings in rodent hippocampal CA1 slices under synaptic blockade, we show that following a brief current pulse, many neurons exhibit exponentially decaying firing rates with a broad distribution of time constants extending to tens of seconds. This indicates that single neurons possess intrinsic mechanisms capable of covering behaviorally relevant temporal scales. These results highlight that single neurons can encode temporal information over more than an order of magnitude, providing a potential cellular substrate for temporal coding in the brain.

## 1 Introduction

Our ability to remember what happened and when is critical for learning temporal relationships and predicting future outcomes. A wealth of evidence implicates the hippocampal formation as a central hub for encoding and retrieving temporal information.

Seminal *in vivo* studies identified hippocampal “time cells”, neurons that fire sequentially after salient events, providing a timeline for temporally organized memories (Eichenbaum, 2014; MacDonald et al., 2011; Pastalkova et al., 2008). Complementing this sequential activity, neurons named “temporal context cells” in the lateral entorhinal cortex (LEC), a region projecting to the hippocampus, were found to exhibit gradually changing firing patterns (Tsao et al., 2018; Bright et al., 2020).

While both sequential time cells and gradually changing temporal context cells are well-established in the behaving animal, the underlying mechanisms that enable neurons to represent time far beyond the duration of a stimulus remain unresolved. In particular, it is unclear whether these temporal dynamics necessitate only network-level interactions or if intrinsic properties of individual neurons are also involved.

Computational work suggests that network mechanisms based on recurrent activity (e.g., reservoir or attractor dynamics) can give rise to both temporal context cells and sequential time cells (Zylberberg and Strowbridge, 2017; Masse et al., 2019; Voelker and Eliasmith, 2018). Modeling work also suggests that single cells could intrinsically generate exponentially decaying firing rates to encode elapsed time (Tiganj et al., 2015; Liu et al., 2019). A population of such neurons with a spectrum of decay rates could be linearly transformed to produce the sequential activity characteristic of time cells, akin to an inverse Laplace transform (Shankar and Howard, 2012; Howard et al., 2014; Tano et al., 2020).

The biological plausibility of an intrinsic cellular mechanism is supported by *in vitro* studies demonstrating that, in the presence of synaptic blockers, individual neurons in the hippocampus and entorhinal cortex can generate persistent firing following a brief stimulus, often induced by cholinergic modulation (Klink and Alonso, 1997; Fransén et al., 2006; Yoshida and Alonso, 2007). However, it is not well understood whether this intrinsically sustained activity can carry graded temporal information. Specifically, it remains unknown whether single neurons can produce gradually decaying firing rates across the wide range of timescales relevant for temporal coding observed in vivo. Such long timescales could play an important role in bridging events in behavioral tasks.

To address this gap, we investigate whether individual hippocampal CA1 neurons, isolated from their network, can intrinsically encode temporal information. We used *in vitro* whole-cell recordings in the presence of synaptic blockers and the cholinergic agonist carbachol (CCh). Neurons were stimulated with brief current injections to induce persistent firing. We demonstrate that many individual CA1 neurons exhibit a gradually decaying firing rate that is well-described by an exponential function. Critically, the time constants of this decay span a wide, continuous range, from seconds to tens of seconds. These results suggest that single neurons possess intrinsic mechanisms to encode temporal information over behaviorally relevant scales, offering a fundamental cellular substrate for memory-encoding circuits.

## 2 Methods

### 2.1 Animals

The experiments were performed at the Ruhr University Bochum, Bochum, Germany, between 2011 and 2014. Experiments were conducted following the guidelines of the local animal ethics committees and the European Communities Council Directive of September 22, 2010 (2010/63/EU). All experimental protocols were approved by the local ethics committee (Der Tierschutzbeauftragte, Ruhr-Universität Bochum). 14–24 days old male and female Long-Evans rats were used to collect data from hippocampal CA1, using three different concentrations of CCh (5, 10, 20μM). The protocols have been previously described in detail (Knauer and Yoshida, 2019).

### 2.2 Acute brain slice preparation

The rats were deeply anesthetized with an intraperitoneal injection of a ketamine:xylazine (100:4 mg/kg) cocktail. Transcardial perfusions with ice-cold cutting solution containing (in mM) 110 choline chloride, 1.25 NaH_2_PO_4_, 7 MgCl_2_, 2.5 KCl, 7 D-Glucose, 3 pyruvic acid, 1 ascorbic acid, 26 NaHCO_3_, and 0.5 CaCl_2_ were performed. The brains were quickly removed from the cranial cavities and immersed in ice-cold cutting solution. 350μm horizontal slices were obtained using a vibratome (VT1000 S, Leica Instruments). Brain slices were transferred into a holding chamber filled with artificial cerebrospinal fluid (ACSF) containing (in mM) 125 NaCl, 1.2 NaH_2_PO_4_, 1.8 MgSO_4_, 3 KCl, 10 D-glucose, 26 NaHCO_3_, and 1.6 CaCl_2_. The slices were subsequently held for 30 min at 30 °C. The pH of the cutting solution and the ACSF were constantly maintained at 7.4 by saturation with 95% O_2_–5% CO_2_. Slices were kept at room temperature for at least 30 min before recording.

### 2.3 Recording

The slices were then transferred to a recording chamber perfused with oxygenated ACSF (35 ± 1 °C) supplemented with kynurenic acid (2mM) and picrotoxin (0.1mM) to block fast ionotropic glutamate and ${\text{GABA}}_{A}$ synaptic transmission, respectively. The cells were visualized using an upright microscope (Axioscope, Zeiss) equipped with a 4x objective, a 40x water-immersion objective, and a monochrome camera (WAT-902H Ultimate, Watec) connected to a computer. The CA1 region was targeted using the 4x objective lens, and the cells were identified with the 40x lens. Patch pipettes (3-8MΩ) were pulled from borosilicate glass capillaries (GB150-8P, Science Products) using a P-87 horizontal puller (Sutter Instruments). The pipettes were back-filled with filtered intracellular solution containing (in mM) 120 K-gluconate, 10 HEPES, 0.2 EGTA, 20 KCl, 2 MgCl_2_, 7 PhCreat di(tris), 4 Na_2_ATP, 0.3 Tris-GTP and 0.1% biocytin for post-hoc location verification (pH adjusted to 7.3 with KOH). The whole-cell patch configuration was achieved by forming a tight seal (≥1GΩ) on the somata of the cells, and electrical access was achieved by applying negative pressure. Electrical signals were amplified using an AxoClamp-2A amplifier (Axon Instruments) and a Multiclamp 700 B (Axon Instruments). The signals were sampled at 20 kHz and low-pass filtered at 10 kHz. Recordings were obtained in current-clamp mode using Clampex 9.0 data acquisition software. The liquid junction potential was not corrected.

### 2.4 Induction of persistent firing

Prior to inducing persistent firing, the membrane potential was adjusted to slightly below the level where spontaneous spikes occur using DC current injection while the cholinergic receptor agonist carbachol (5, 10 or 20μM) was continuously bath applied (Knauer et al., 2013). Persistent firing was then induced using a brief current injection (2s,100pA). The membrane potential recorded after the offset of the brief current injection was used to analyze persistent firing in the following sections.

### 2.5 Data analysis

We first transformed the raw membrane-potential recordings into instantaneous firing rates. An action potential was identified when the membrane potential surpassed a threshold of -10mV, indicating a depolarization event. Binned firing rates were computed as the number of spikes occurring within 1s windows, advanced in 100ms steps.

To characterize gradual changes in neuronal activity following the current injection, we fitted exponential and linear functions to the binned firing rate. Specifically, to test the hypothesis about exponentially changing firing rate, we modeled the firing rate of individual neurons using the following model:

(1) $${\overset{ˆ}{y}}_{e}\left(t_{i}\right)=a_{0}+a_{1}e^{-t_{i}/\tau },$$

with parameters $a_{0}$ and $a_{1}$ capturing the magnitude of the firing rate, and τ capturing the time constant. To keep $\overset{ˆ}{y}$ strictly positive, we constrained the parameters such that $a_{0}>0$ and $a_{0}+a_{1}>0$.

To evaluate whether linearly changing firing rate could better describe the neuronal activity, we fitted the firing rate with the following model:

(2) $${\overset{ˆ}{y}}_{l}\left(t_{i}\right)=wt_{i}+c,$$

that included slope parameter w and intercept parameter c. We also computed a fit with a constant term ${\overset{ˆ}{y}}_{c}\left(t_{i}\right)=c$, which captures neurons that responded to the current injection with stable persistent firing.

We fit 427 bins (≈ 43.5 s) beginning at the offset of the current pulse. The parameters were optimized to reduce root mean square error (RMSE) between $\overset{ˆ}{y}$ and the true firing rate (t):

(3) $$L=\sqrt{\frac{1}{N}\sum_{i=1}^{N}{\left(\hat{y}\left(t_{i}\right)-y\left(t_{i}\right)\right)}^{2}}\text{,}$$

where N=427 is the number of time bins, $t_{1}=0\;s$ and $t_{427}\approx 43\;s$. Curve-fitting was done using the Python package *lmfit*.

For each cell, we compared adjusted $R^{2}$ between the exponential, linear and constant models:

(4) $$R_{\text{adj}}^{2}=1-\left(\frac{\left(1-R^{2}\right)(n-1)}{n-k-1}\right),$$

where n is the number of time bins and k is the number of free parameters (k=3 for exponential model, k=2 for linear model and k=1 for the constant model) and $R^{2}$ is coefficient of determination:

(5) $$R^{2}=1-\frac{\sum_{i=1}^{n}{\left(y\left(t_{i}\right)-\overset{ˆ}{y}\left(t_{i}\right)\right)}^{2}}{\sum_{i=1}^{n}{\left(y\left(t_{i}\right)-\overset{‾}{y}\right)}^{2}}.$$

To evaluate the robustness of the temporal changes in the persistent firing of exponentially decaying cells, we estimated the elapsed time since the current injection from each neuron:

(6) $$\overset{ˆ}{t}=\tau ln\left(\frac{a_{1}}{y-a_{0}}\right).$$

## 3 Results

**Figure 1:**
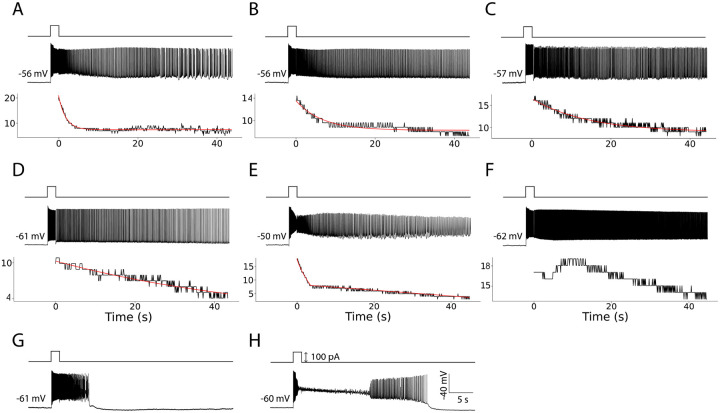
Examples of neurons with: **A.-C.** Exponentially decaying firing rate, sorted by time constant from smallest (left) to largest (right). **D.** A firing rate best fit with a linear function. **E.** A firing rate best fit with a combination of linear and exponential fits. **F.** Maximum firing rate occurring after 5s. **G.** Self-terminating persistent firing H. Depolarization block. In each panel, the top row shows the time when the current was injected, the second row shows raw membrane potentials, and the third row (if applicable) shows firing rate (Hz) (black lines) and the best fit with either an exponential or a linear function (red lines).

We analyzed *in vitro* activity from 98 CA1 pyramidal neurons across three different CCh concentrations following a current pulse injection. We focused on neurons with sustained gradually changing firing rates, which did not self-terminate for at least 30s following the pulse injection (Fig. 1A–F).

We considered that depolarization block had occurred when the membrane potential entered a sustained depolarized plateau above the cell’s physiological spike threshold, accompanied by a loss of over-shooting spikes (i.e., no events crossing -10mV) for more than 5s after current injection. These criteria for self-termination and depolarization block were adopted from Brahimi et al. (2023). We did not observe any neurons for which the fit with a constant term was better than the fit with either an exponential or a linear model. Thus, out of 98 neurons, the firing activity of 67 (68%) neurons was best fit with models that include gradual change across time.

### 3.1 Exponentially decaying firing was the most commonly observed activity profile

Among the 67 remaining neurons with gradually changing firing activity, 32 neurons (48%) were best fit with the exponential decay (Fig. 1A–C). We note that in all cases where the exponential model was better than others, the exponent was always negative, indicating a decaying firing rate. We also observed 11 neurons (16%) where the linear fit was better than the exponential one (Fig. 1D, Table 1), also with a negative slope indicating again a decaying firing rate.

A subset of 24 neurons was poorly described by both exponential and linear models and was therefore excluded from these counts and the subsequent analysis. Specifically, some neurons whose activity was nominally best captured by the exponential model exhibited two phases: a steep decline during the first ~5s followed by a slower decay. While the exponential fit was better than the linear or constant fit for such cells, it was overall poorly capturing the firing dynamics (Fig. 1E). To identify these cells, we separated the neuronal activity into two sections, before and after the first 5s, and compared the adjusted $R^{2}$ for a combination of linear and exponential fits for each section. This new fit had a higher adjusted $R^{2}$ (with 5 parameters) than the exponential fit for 12 neurons (17%). In addition, 12 neurons were fitted best with decaying functions, but had a temporary increase in firing activity that peaked more than 5s after the current injection (Fig. 1F). We consider the firing rate of these neurons as non-monotonically changing rather than gradually increasing or decreasing. Activity of all analyzed neurons together with exponential and linear fits is shown in the Appendix.

Of the 98 neurons, 31 of them did not show such persistent firing activity due to either self-terminating activity (Fig. 1G, 15 neurons) or depolarization block (Fig. 1H, 16 neurons) (Table 1). Since neurons whose activity was best fit with the exponentially decaying functions were by far most frequent, they were the focus of our subsequent analysis, where we further investigated their firing properties.

### 3.2 Diversity of time scales: exponential decay spans a range of time constants extending to tens of seconds

**Figure 2:**
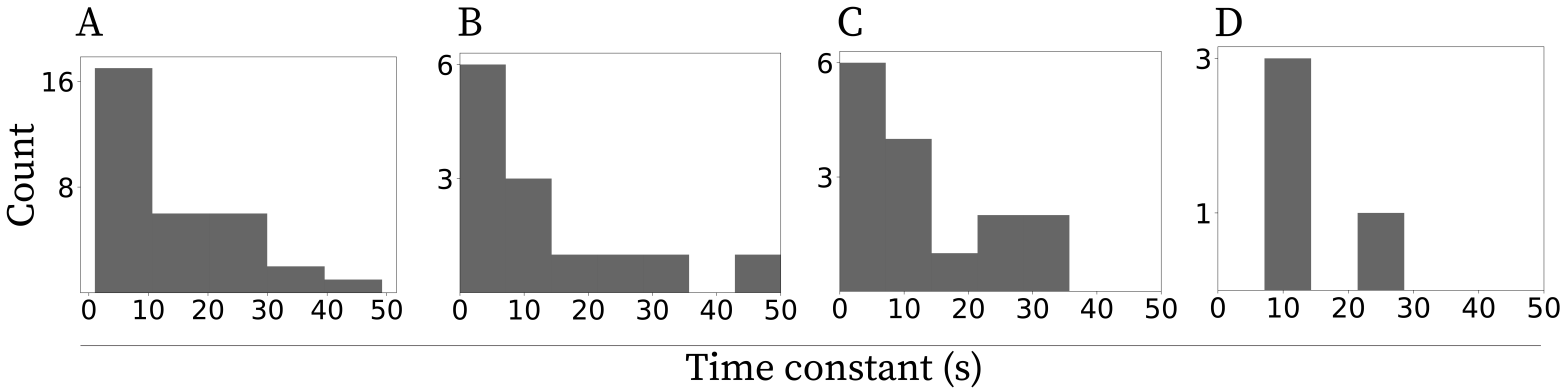
Neurons with exponentially decaying firing profiles span a wide range of temporal scales. Histograms of time constants indicate a decreasing number density of neurons as a function of the time constant. **A.** Neurons from all three CCh concentrations combined. **B.** 5μM CCh. **C.** 10μM CCh. **D.** 20μM CCh.

We observed a broad range of temporal scales for neurons whose activity was best fit with the exponentially decaying model. The distribution of fitted time constants is shown in Fig. 2A. Fig. 2B–D show the histograms for each of the three CCh concentrations. The time constants overall ranged from 1.1s to 49.3s. To provide a better sense of the activity profiles, plots in the Appendix show activity of all 32 neurons together with the exponential fits sorted by the time constant of the fit (for illustrations see Fig. 1A–C for neurons fitted with exponentially decaying functions with time constants of 1.81s,6.61s and 14.12s respectively). In general, the number density of neurons decreased as a function of time constant, contributing to the gradual decrease of temporal resolution following the current injection. We found no effect of carbachol concentration on the decay time constants. A Kruskal-Wallis test showed no significant difference among the three groups (5, 10, and $\left.20\;\mu \text{M};H(2,N=32)=0.41,p=0.81,\eta^{2}=0.02\right)$. However, we note that the proportion of neurons exhibiting such decay was significantly lower at 20μM (Table 1), suggesting that while higher cholinergic drive reduces the prevalence of this coding regime, it may not alter the temporal dynamics of the cells that remain within it.

### 3.3 Higher CCh concentration shifts the distribution of activity profiles away from exponential decay and toward DB and/or ST

We compared the proportion of CA1 neurons whose firing was best fit with the exponentially decaying model across three carbachol concentrations (5, 10, and 20μM; 25, 39, and 34 neurons, respectively). A $\chi^{2}$ test of independence revealed a significant association between concentration and decay type, $\chi^{2}(2,N=98)=11.60,p=0.0030$, Cramér’s V=0.34 (moderate effect).

Pairwise Fisher exact tests (Holm-corrected) showed that the 20μM group had significantly fewer exponentially decaying neurons than both 5μM (odds ratio = 8.1, $p_{\text{corr}}=0.0035$) and 10μM (odds ratio = 4.7, $p_{\text{corr}}=0.030$), whereas the difference between 5μM and 10μM was not significant ($p_{\text{corr}}=0.31$). Overall, our results indicate that exponentially decaying firing is more prevalent at lower CCh concentrations. Beyond the reduction in exponentially decaying neurons, higher CCh also shifted response outcomes toward failure modes. At 20μM, the proportion of depolarization block (DB) rose to 38.2% (13/34) compared with 4.0% (1/25) at 5μM and 5.1% (2/39) at 10μM; self-terminating (ST) responses likewise increased to 23.5% (8/34) vs. 12.0% (3/25) and 10.3% (4/39). Fisher’s exact tests comparing 20μM to pooled 5-10μM indicated substantially higher odds of DB (odds ratio OR = 12.59, $p=4.39\times 10^{-5}$) and a moderate, non-significant increase for ST (OR = 2.51, p=0.140). These patterns suggest that excessive cholinergic drive increases the likelihood that persistent firing either collapses (ST) or enters a depolarized, spike-incompetent state (DB).

### 3.4 Exponential decay can enable estimation of elapsed time

**Figure 3:**
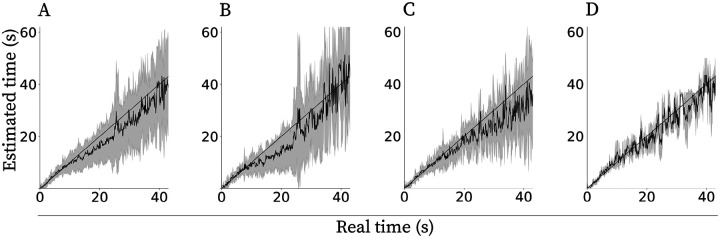
Estimating elapsed time from exponentially decaying cells. Note that the standard deviation of the estimate (gray lines) increases with time. A solid straight line represents a perfect estimate and a solid curvy line is the mean estimate across cells. **A.** Neurons from all three CCh concentrations combined. **B.** 5μM CCh. **C.** 10μM CCh. **D.** 20μM CCh.

Figure 3 shows estimation of elapsed time from the exponentially decaying neurons. The end of the current injection is considered time zero. The estimation was done for each neuron with exponentially decaying firing rate following Eq. 6. We then averaged the estimated time from all neurons from the same CCh condition and compared it to the actual elapsed time. Consistent with Weber’s law, the standard deviation of the estimate increased with elapsed time. The correlation between standard deviation of the estimated time and the real time was found to be significant for all conditions (for 5μM CCh r=0.849,p<0.001, for 10μM CCh r=0.909,p<0.001, for 20μM CCh r=0.556,p<0.001). Note that since this analysis was conducted with a different number of neurons per condition, the statistical power in each condition was rather different (Table 1), accounting for the large variability observed across conditions.

### 3.5 Anatomical position and age were not detectably related to decay time constants

**Figure 4:**
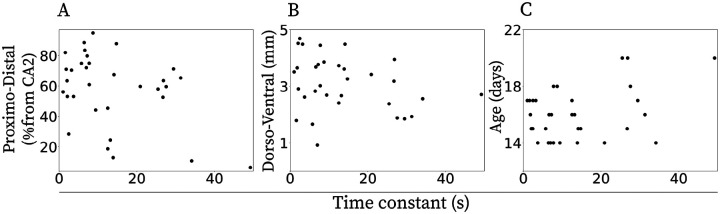
Relationship between the estimated time constant and anatomical locations and age of the neurons with exponentially decaying firing rates for all CCh concentrations combined. **A.** Proximo-distal distance (% from CA2), **B.** Dorsal-ventral distance, and **C.** Age of the animal. No significant correlation was observed.

To test whether decay time constants vary systematically with cell location and age, we pooled neurons across all CCh concentrations and correlated each neuron’s time constant with its dorsal–ventral and proximo–distal coordinates (Figure 4) as well as animals’ age (14–24 days). After Bonferroni correction across three comparisons, Pearson coefficients were not significant. Specifically for dorsal–ventral: r=-0.28,p=0.30,n=32, for proximo–distal: r=-0.40,p=0.06,n=32 and age r=0.36,p=0.04,n=32. Together, these analyses indicate that neither recording depth, proximo–distal position, nor age measurably influences the range of decay time constants observed in CA1 neurons under our conditions.

Additionally, we examined whether parameters measured during the induction of persistent firing correlated with the decay time constant. Across all CCh concentrations, no significant correlations were found with baseline membrane potential (BL), defined as the mean membrane potential during the 1s before stimulus onset (r=-0.28,p=0.12,n=32), or with the holding current during BL (r=0.15,p=0.40,n=32). We used 1s intervals to reduce moment-to-moment noise while preserving temporal resolution. Similarly, the decay constant did not correlate with the difference between BL and the final steady state membrane potential (mean membrane potential during the last 1s of the recording; r=-0.16,p=0.37,n=32), nor with the difference between the immediate post-stimulus potential (mean membrane potential during the 1s after stimulation) and the final steady state membrane potential (r=-0.16,p=0.37,n=32).

Given the small per-condition samples, these correlation estimates are imprecise and our null findings should be interpreted cautiously.

## 4 Discussion

In this study, we report and characterize single-neuron persistent firing in hippocampal CA1 neurons in rats in the presence of synaptic blockers and CCh. Our principal finding is that, following a brief depolarizing current injection, many neurons exhibit gradually decaying firing rates spanning a wide range of time constants (approximately 1 s to 50 s). Note that the upper edge of the time constants distribution was limited by the recording duration. These time constants occupy behaviorally relevant scales and fall within the temporal regime relevant for time cells (Eichenbaum, 2014; MacDonald et al., 2011; Pastalkova et al., 2008; Howard and Eichenbaum, 2013) and temporal context cells (Tsao et al., 2018; Bright et al., 2020). This illustrates that even a single-cell mechanism could give rise to these firing profiles.

The observation that neural firing was not characterized by a stable firing rate, but by gradually changing activity, indicates that even in the presence of synaptic blockers, neurons encoded temporal information. In the absence of synaptic blockers, some of these neurons would presumably receive inputs that are modulated by the identity of the input stimuli. Coupled with the temporal modulation, this would enable simultaneous coding of “what” happened “when” in the working memory of the recent past. Neurons selective to stimulus identity and elapsed time have been reported in monkey prefrontal cortex and hippocampus during the delayed-match-to-sample task (Tiganj et al., 2018; Cruzado et al., 2020) and in bat hippocampus during social exploration (named “contextual time cells”) (Omer et al., 2023).

### 4.1 Multi-scale exponential decay as a basis of computational models of memory and learning

We focused our analysis on exponentially decaying firing activity due to its importance in computational models of memory and learning. Exponentially decaying temporal context is a basis of the Temporal Context Model (TCM) (Howard and Kahana, 2002) and its generalized version, Context Maintenance and Retrieval (CMR) model (Polyn et al., 2009). While these models rely on a single time constant, their extensions use exponentially decaying impulse responses with a spectrum of time constants to approximate an integral transform of the input. This enables encoding of temporal patterns rather than single stimulus presentations and gives rise to power-law forgetting, which naturally emerges as an integral of multi-scale exponential functions (Shankar and Howard, 2012; Howard et al., 2015).

Critically, the ingredient for power-law forgetting in the framework from Shankar and Howard (2012) and Howard et al. (2015) is that the distribution of time constants decreases as a power-law function. While the number of cells in our analysis is insufficient to argue for a particular distribution of time constants (Fig. 2A), we have illustrated that the distribution is not uniform and that the number density decreases as a function of time constant, supporting the gradual decay of memory.

If individual neurons operate in a regime where they can be considered linear systems, a population of such neurons can encode an integral transform of the recent past. A linear system would be characterized by an impulse response: the neuronal activity would be a convolution of the input signal (current injection) with the impulse response. While we established the existence of neurons with a wide range of time constants, we were unable to test whether the exponential decay can be considered the impulse response of these neurons. Since we used a single current pulse of fixed amplitude, we were unable to test the linearity of the response. Future work could investigate this by systemically manipulating the input, as well as CCh concentration, to determine whether these neurons operate within a linear regime. If single neurons truly behave as linear filters with exponential kernels, a heterogeneous population could encode rich temporal patterns. Such a mechanism could underlie a working-memory trace of “what happened when”.

In model-free and model-based reinforcement learning, efficient recursive computation of expected future reward is enabled through the Bellman equation, which requires exponential temporal discounting (Sutton and Barto, 2018; Dayan, 1993). Models with a spectrum of time constants show that integrating the exponentially discounted values leads to human-like power-law and hyperbolic discounting (Sozou, 1998; Kurth-Nelson and Redish, 2009; Tiganj et al., 2019; Momennejad and Howard, 2018; Tano et al., 2020). Exponentially decaying neurons could thus supply a substrate for encoding temporally extended value functions and guiding multi-step planning, adapting neural circuits to tasks that demand flexible action selection over a range of temporal windows.

### 4.2 Impact of CCh concentration on neural time constants

Despite significant differences across CCh concentrations, we always observed some neurons whose activity was best fit with exponentially and linearly decaying functions (Table 1). The proportion of neurons with exponentially decaying firing decreased when increasing carbachol from 5μM to 20μM. One possibility is that while muscarinic receptor activation is critical for triggering persistent firing, excessive cholinergic stimulation may shift neurons toward a depolarization block or a saturated conductance state (Knauer and Yoshida, 2019). At moderate concentrations (5-10μM), the interaction between inward current that depolarized the membrane to drive persistent firing (e.g., TRPC current) and outward currents, which counteract the depolarization (e.g., SK and M currents) might have resulted in a gradually decaying persistent firing in many cells (Klink and Alonso, 1997; Egorov et al., 2002). As CCh levels rise further, however, additional or amplified inward currents may overwhelm homeostatic mechanisms, leading to prolonged depolarization and eventual block of spike generation (Tai et al., 2011; Knauer and Yoshida, 2019). Consequently, the fine-tuned balance required for a stable, exponential decay is absent in a larger fraction of neurons at 20μM.

An alternative or complementary explanation involves competing ionic or signaling processes that become dominant only under higher cholinergic drive. The same channel populations or second-messenger systems that support persistent firing at moderate CCh levels may saturate at higher concentrations. In turn, neuronal excitability and repolarization may fail to transition through the narrow parameter regime necessary for an exponential decay profile (Fransén et al., 2006). Hence, although cholinergic modulation is critical for persistent firing, these data highlight a potential nonmonotonic relationship between the degree of muscarinic receptor activation and the fraction of neurons expressing smoothly decaying activity.

### 4.3 Phasic cholinergic signals as event tags for encoding

In behaving animals, cholinergic activity exhibits brief, cue-locked transients that are tightly linked to whether a stimulus is detected: prefrontal acetylcholine release rises on hit trials, and bidirectional optogenetic manipulations show that suppressing these transients impairs detection, whereas generating them can increase false alarms (Parikh et al., 2007; Gritton et al., 2016). Beyond detection, these events coincide with rapid shifts in local dynamics—elevated gamma power and theta-gamma coupling during cue epochs—consistent with a fast state-switching influence on cortical processing (Howe et al., 2017). Although the precise computational role of such phasic signals remains debated (Sarter et al., 2016), their millisecond-to-second timing and trial specificity support the view that they mark behaviorally significant moments (“event boundaries”) and open brief windows of heightened excitability and plasticity.

Extending this view to hippocampal memory circuits, coordinated cholinergic release has been observed across prefrontal cortex and hippocampus on distinct timescales associated with arousal and reward, providing a route for aligning attentional and mnemonic operations around salient events (Teles-Grilo Ruivo et al., 2017). Within the hippocampal formation, cholinergic modulation biases networks toward encoding by enhancing the impact of afferent inputs and facilitating synaptic modification (Hasselmo, 2006; Teles-Grilo Ruivo and Mellor, 2013). This distinction is further supported by recent human intracranial recordings showing that muscarinic blockade with scopolamine impairs memory when present during encoding, but does not significantly impair performance when restricted to retrieval (Gedankien et al., 2025). Taken together, these findings are consistent with the possibility that brief cue-evoked cholinergic bursts could initiate or reset the intrinsic exponential decays expressed in CA1 under muscarinic activation, effectively “stamping” discrete events with a temporal tag while coordinating cortical—hippocampal processing to bind what happened to when it happened (Parikh et al., 2007; Gritton et al., 2016; Howe et al., 2017; Teles-Grilo Ruivo et al., 2017; Hasselmo, 2006; Teles-Grilo Ruivo and Mellor, 2013; Sarter et al., 2016).

### 4.4 Integration with extrahippocampal signals and behavioral state

Although we tested these neurons in a controlled slice preparation, the wide distribution of decay time constants across cells and conditions suggests a natural substrate for flexible temporal coding in the intact brain. Indeed, recent in vivo evidence supports the involvement of intrinsic persistent firing in working memory (Saber Marouf et al., 2025). In behaving animals, external inputs from frontal or parahippocampal regions could reset or modulate a neuron’s exponential decay, thus linking intrinsic cellular timescales with ongoing behavioral states (e.g., exploration, alertness, or consummatory behavior). This interaction may be particularly important when the task involves contextual changes on multi-second or minute-long intervals; exponential decays at different rates could quickly adapt to reflect new reference points for “time since event”, allowing hippocampal circuits to anchor retrieval processes or prospective encoding. Future experiments combining selective neuromodulatory manipulations and *in vivo* recordings during memory tasks would help reveal how internal dynamics, evident even in isolated neurons, interface with network-level processes to govern the timing of hippocampal-dependent behaviors.

## Supplementary Material

Supplement 1

## Figures and Tables

**Table 1:** Response types of CA1 pyramidal neurons at three CCh concentrations. Numbers indicate counts of neurons in each category.

CCh (μM)	# Total	# DB	# ST	# Peak	# Two-phase	# Lin. dec.	# Exp. dec.
5	25	1	3	2	3	3	13
10	39	2	4	6	7	5	15
20	34	13	8	4	2	3	4

**DB**, depolarization block; **ST**, self-terminating within 30 s; **Peak**, delayed peak (> 5 s after pulse); **Two-phase**, biphasic decay with a rapid initial drop followed by a slower phase. **Lin. dec**., linearly decaying firing; **Exp. dec.**, exponentially decaying firing;
