# Supplementary material for: Intrinsic persistent firing in CA1 encodes elapsed time across behaviorally relevant scales
Source: bioRxiv. 2025 Dec 13:2025.12.10.693586. Preprint. [Version 1] doi: 10.64898/2025.12.10.693586 (PMC12714023; doi:10.64898/2025.12.10.693586)
Supplement: Supplement 1 [file media-1.pdf]

Supplementary material

Neurons with firing rates best fit by an exponentially decaying model in the presence of 5 μM CCh

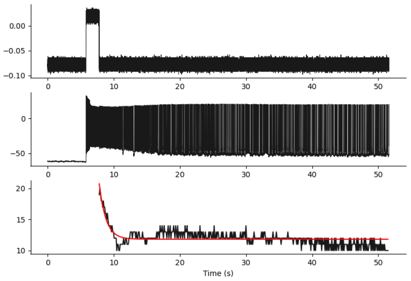

Cell ID: $1, \tau$: 1.02

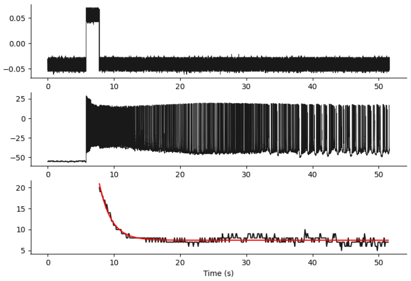

Cell ID: $2, \tau$: 1.81

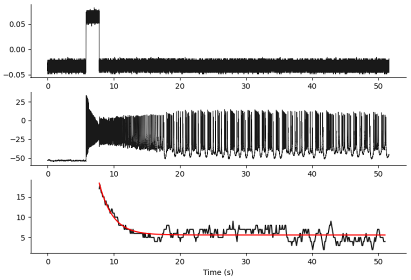

Cell ID: $3, \tau$: 2.04

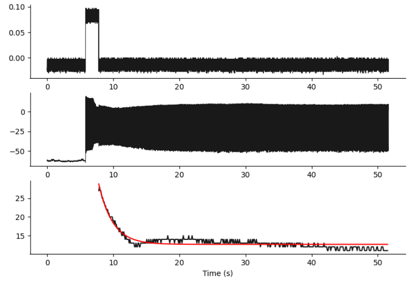

Cell ID: $4, \tau$: 2.11

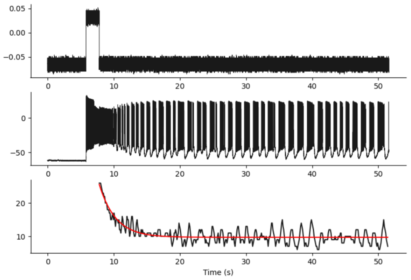

Cell ID: $5, \tau$: 2.47

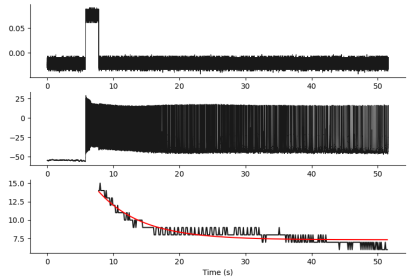

Cell ID: $6, \tau$: 6.61

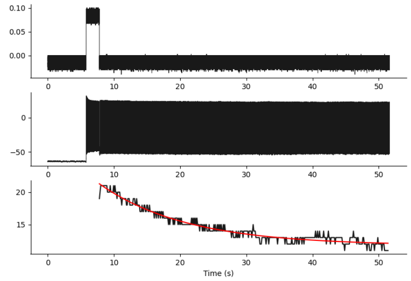

Cell ID: $7, \tau$: 13.18

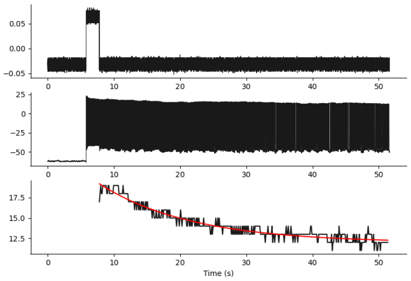

Cell ID: $8, \tau$: 13.90

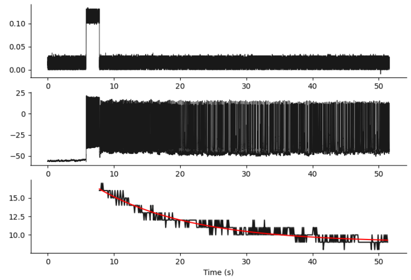

Cell ID: $9, \tau$: 14.11

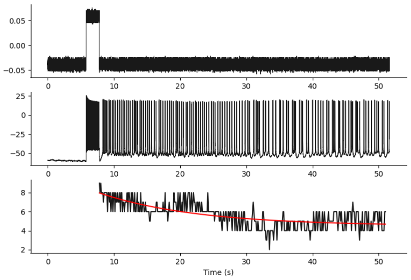

Cell ID: $10, \tau$: 14.74

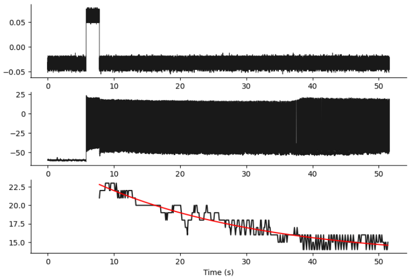

Cell ID: $11, \tau$: 25.45

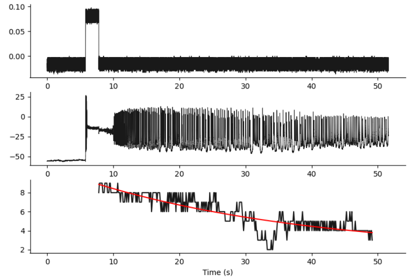

Cell ID: $12, \tau$: 34.11

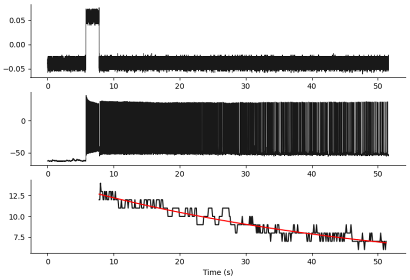

Cell ID: $13, \tau$: 49.22

Neurons with firing rates best fit by an exponentially decaying model in the presence of 10 μM CCh

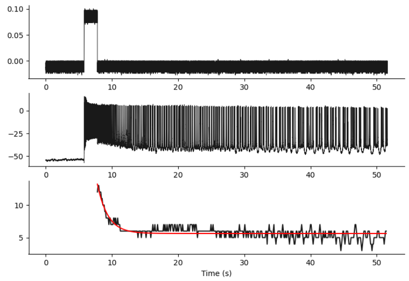

Cell ID: 14, τ: 1.59

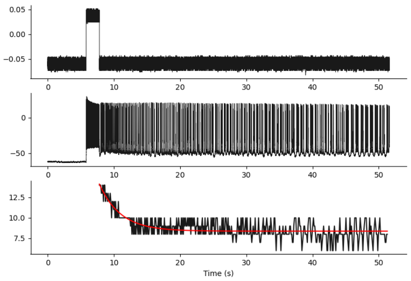

Cell ID: 15, τ: 3.19

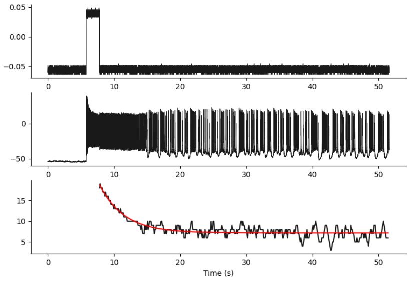

Cell ID: 16, τ: 3.71

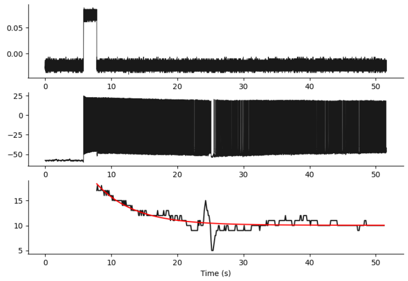

Cell ID: 17, τ: 5.74

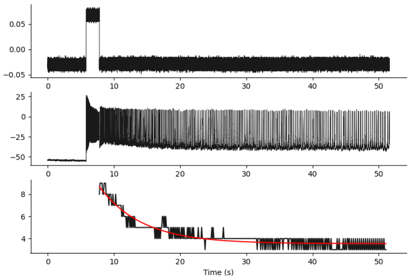

Cell ID: 18, τ: 6.45

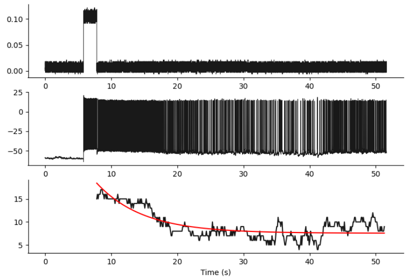

Cell ID: 19, τ: 7.01

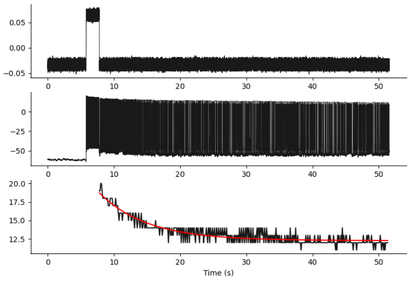

Cell ID: 20, τ: 7.21

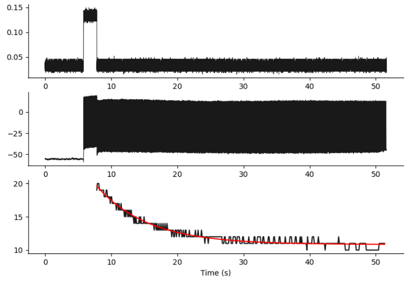

Cell ID: 21, τ: 7.74

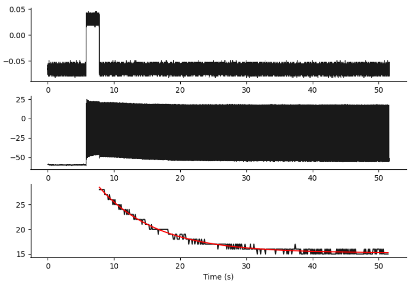

Cell ID: 22, τ: 8.71

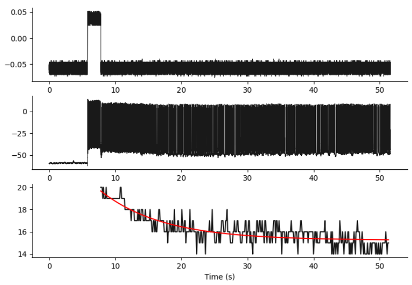

Cell ID: 23, τ: 9.40

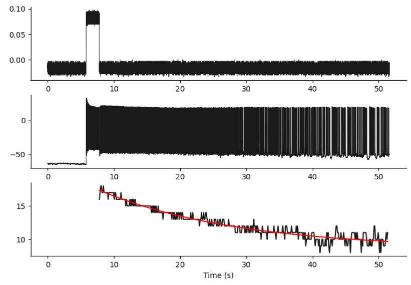

Cell ID: 24, τ: 20.88

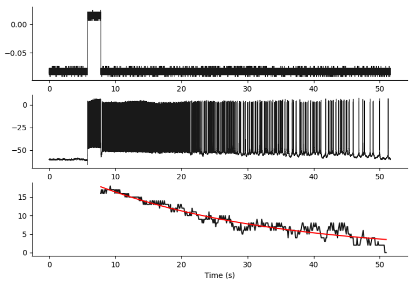

Cell ID: 25, τ: 26.72

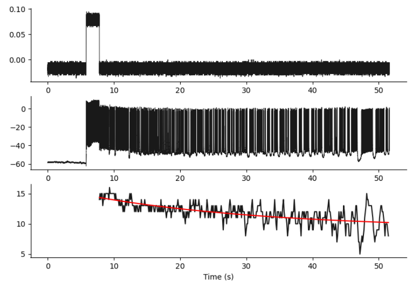

Cell ID: 26, τ: 27.55

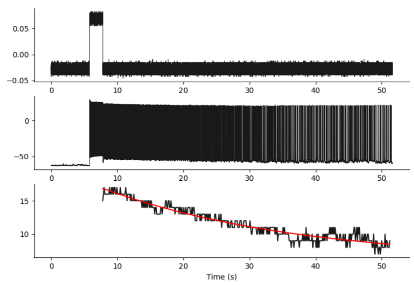

Cell ID: 27, τ: 29.44

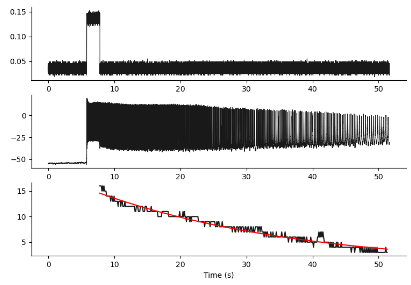

Cell ID: 28, τ: 31.29

Neurons with firing rates best fit by an exponentially decaying model in the presence of 20 μM CCh

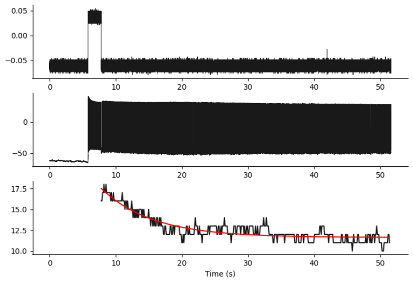

Cell ID: 29, τ: 7.76

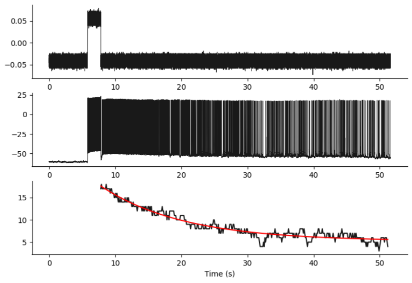

Cell ID: 30, τ: 12.51

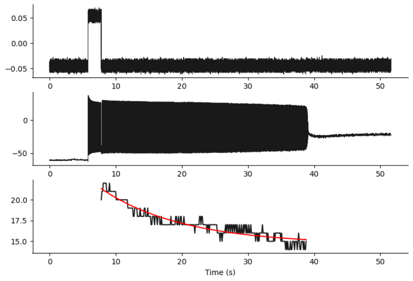

Cell ID: 31, τ: 12.52

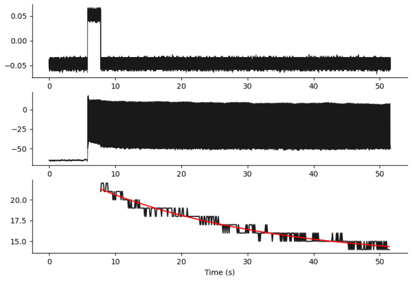

Cell ID: 32, τ: 26.84

Neurons with firing rates best fit by a linear model in the presence of 5 μM CCh

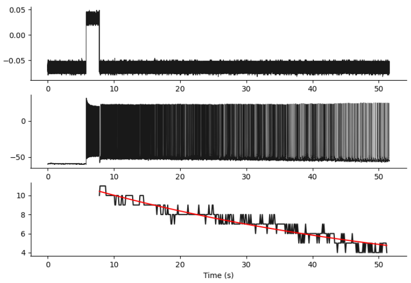

Cell ID: 33, a:-0.132

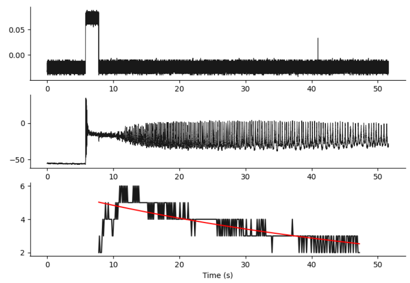

Cell ID: 34, a:-0.066

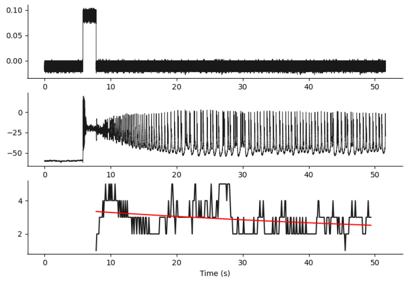

Cell ID: 35, a:-0.02

Neurons with firing rates best fit by a linear model in the presence of 10 μM CCh

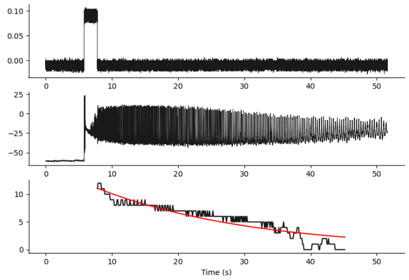

Cell ID: 36, a:-0.257

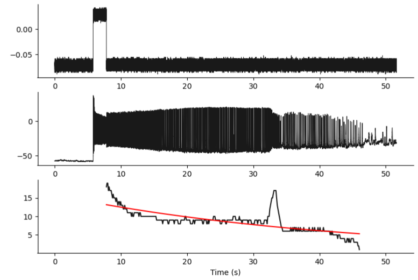

Cell ID: 37, a:-0.207

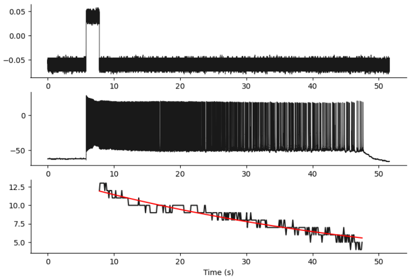

Cell ID: 38, a:-0.161

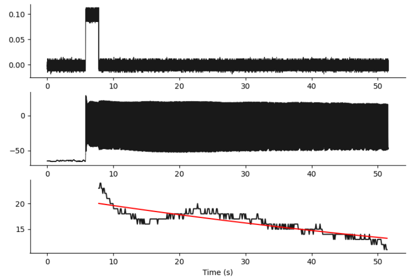

Cell ID: 39, a:-0.159

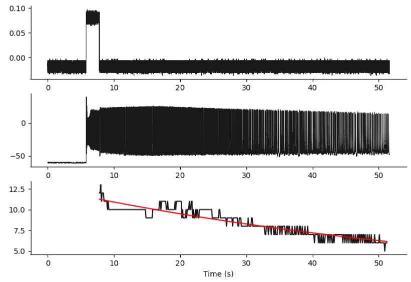

Cell ID: 40, a:-0.119

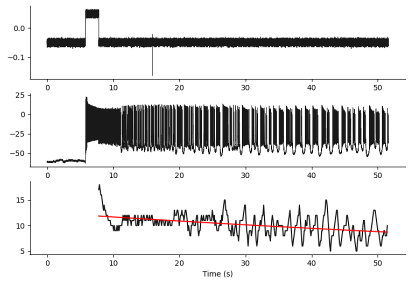

Cell ID: 41, a:-0.072

Neurons with firing rates best fit by a linear model in the presence of 20 μM CCh

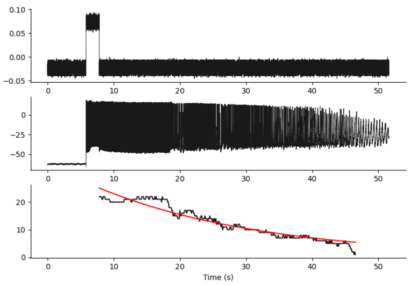

Cell ID: 42, a:-0.523

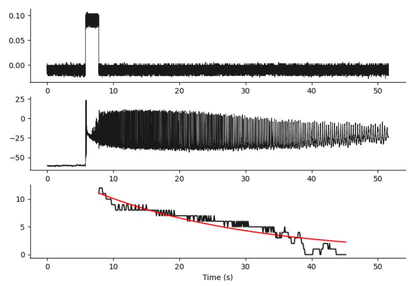

Cell ID: 43, a:-0.257

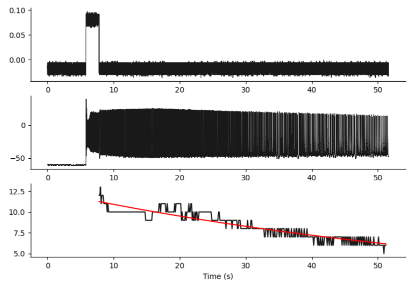

Cell ID: 44, a:-0.119

Neurons with firing rates best by a combination of linear and exponential models

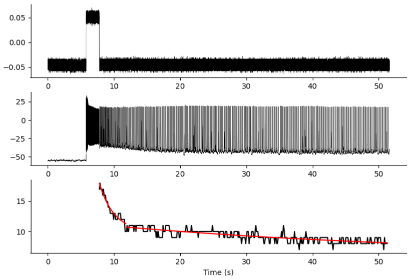

Cell ID: 45, 5 μM CCh

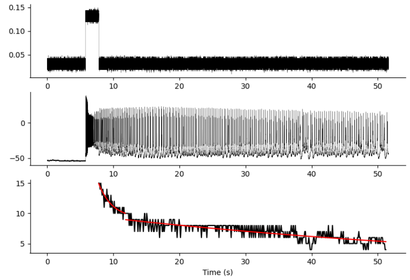

Cell ID: 46, 5 μM CCh

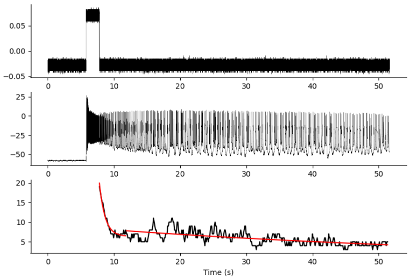

Cell ID: 47, 5 μM CCh

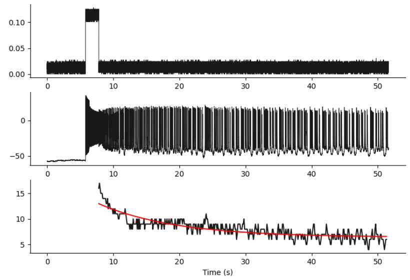

Cell ID: 48, 10 μM CCh

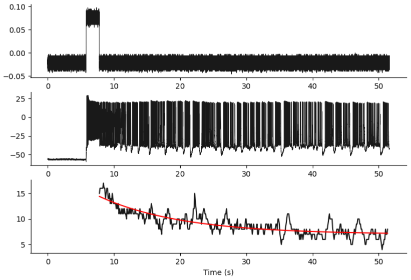

Cell ID: 49, 10 μM CCh

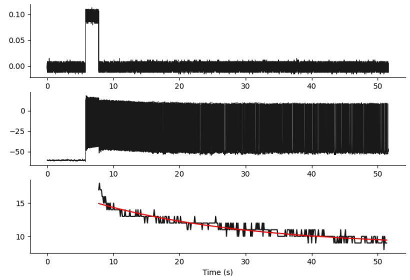

Cell ID: 50, 10 μM CCh

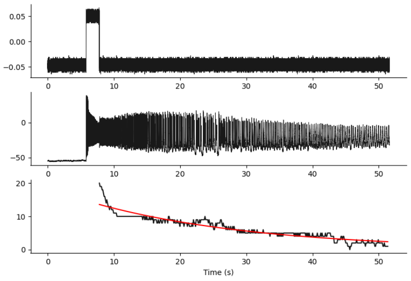

Cell ID: 51, 10 μM CCh

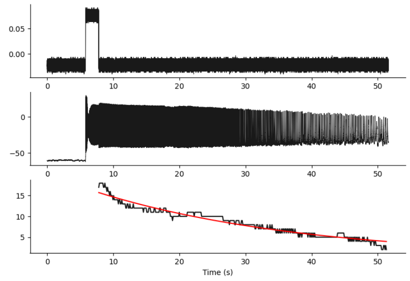

Cell ID: 52, 10 μM CCh

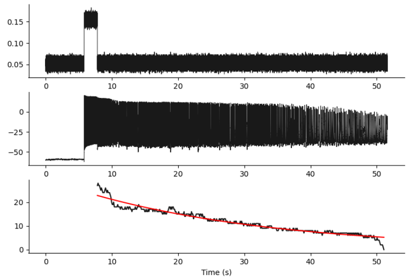

Cell ID: 53, 10 μM CCh

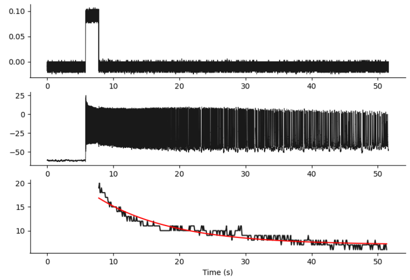

Cell ID: 54, 10 μM CCh

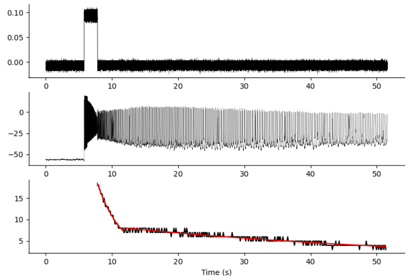

Cell ID: 55, 20 μM CCh

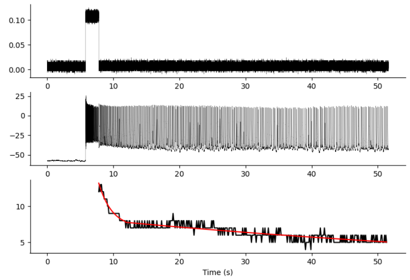

Cell ID: 56, 20 μM CCh

Neurons with maximum firing rate occurring after 5 s

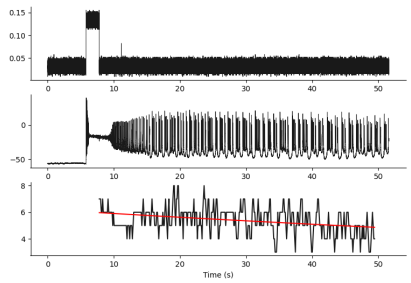

Cell ID: 57, 5 μM CCh

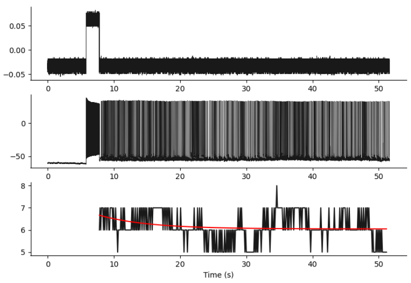

Cell ID: 58, 5 μM CCh

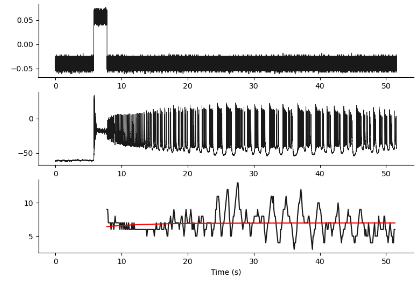

Cell ID: 59, 10 μM CCh

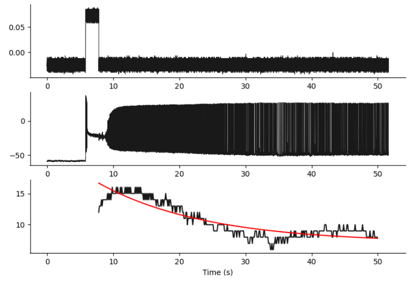

Cell ID: 60, 10 μM CCh

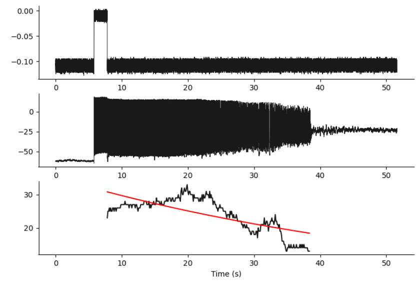

Cell ID: 61, 10 μM CCh

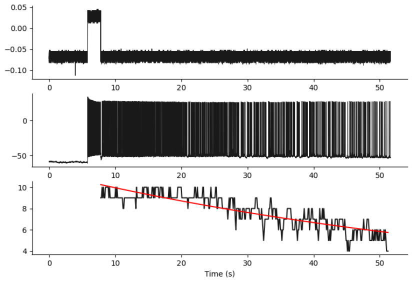

Cell ID: 62, 10 μM CCh

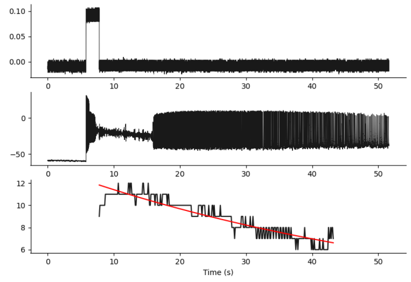

Cell ID: 63, 10 μM CCh

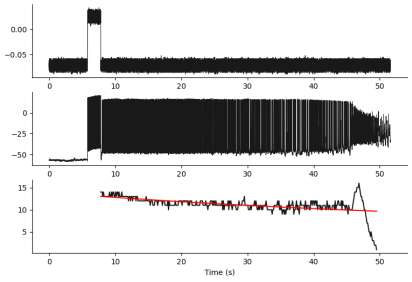

Cell ID: 64, 10 μM CCh

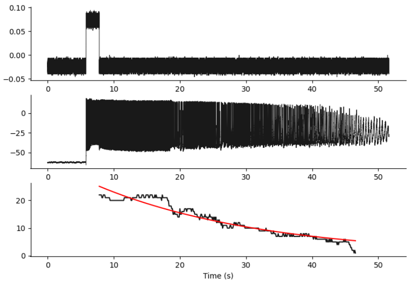

Cell ID: 65, 20 μM CCh

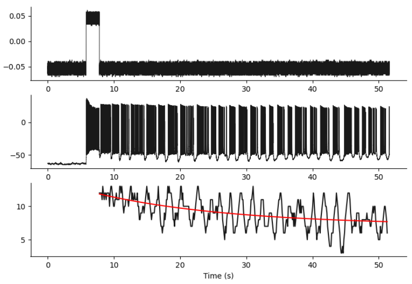

Cell ID: 66, 20 μM CCh

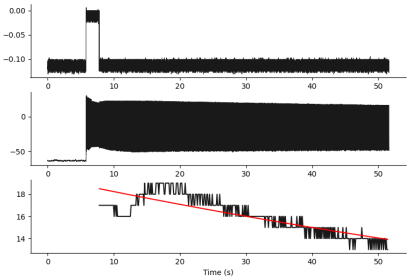

Cell ID: 67, 20 μM CCh

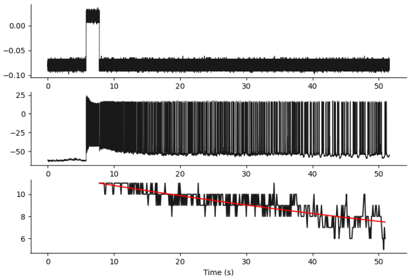

Cell ID: 68, 20 μM CCh
